# Study on the Attitudes and Knowledge of Teachers and Future Teachers about Immediate Health Care Measures at School

**DOI:** 10.3390/ejihpe12070062

**Published:** 2022-07-20

**Authors:** Paula Pais-Roldán, María del Carmen Olmos-Gómez, Jesús Manuel Cuevas-Rincón, Mónica Luque-Suárez

**Affiliations:** 1 Department of Research Methods and Diagnosis in Education, Faculty of Education and Sport Science, University of Granada, 52071 Melilla, Spain; paulapr@correo.ugr.es (P.P.-R.); jcuevas@ugr.es (J.M.C.-R.); 2 Department of Sociology, Faculty of Education and Sport Science, University of Granada, 52071 Melilla, Spain; mlsuarez@ugr.es

**Keywords:** first aid, early childhood and primary education teachers, validation, education

## Abstract

The level and need for immediate health care measures training for teachers are of growing concern for two main reasons: on the one hand, these contents are part of the school curriculum and, on the other hand, teachers are the first adults to intervene in case of school injury. However, in Spain, first aid (FA) does not appear as obligatory content in the university training of teachers. The aim of the present study was to design and validate a questionnaire on the attitude of pre-school and primary school teachers towards first aid knowledge adapted to the school context and to analyze its psychometric properties. First of all, the psychometric values of the questionnaire were tested: through its validation and reliability. Subsequently, a correlation study was carried out as well as a logistic regression in order to know the knowledge and attitudes of teachers and future teachers about the importance of the Immediate Attention Measures at school. The sample consisted of 392 participants: active teachers (71%) and future teachers (29%, the latter being final year students) of the infant and primary stages from the northern, central and southern areas of Spain. The results show adequate psychometric values, establishing three factors: attitude towards general knowledge in FA and learning methodology; attitude towards wounds and CPR algorithm (most frequent and/or serious events); self-perception of knowledge or skill in FA. Pearson’s correlation test identified significant values (*p* < 0.01) and positive association between Factors 1 and 2 (r = 0.422) and between 1 and 3 (0.244). The conclusions of the results of the validation process of the questionnaire on the attitude of teachers and future teachers to knowledge for immediate health care measures training are valid and reliable to an acceptable degree. Regression study demonstrates the importance of including first aid training in teachers’ degrees.

## 1. Introduction

The European Council for cardiopulmonary resuscitation (ERC) defines first aid [1] as the initial care and helping behaviors that anyone in any situation can provide in the face of acute injury or illness and which are intended to preserve life, reduce suffering, prevent injury or complications and/or promote the recovery of victims. In fact, one of the main objectives of the ERC is to promote the teaching of Cardiopulmonary Resuscitation (CPR) among the general population.

The growing concern about health and, more especially, about the health of schoolchildren has led various countries to implement first aid (FA) training at different educational levels and stages; however, there is no consensus in this regard. For example, in 2017 [2], 39 US states and Washington DC included CPR education and training as a requirement for a high school diploma. In countries such as Denmark, Germany, Italy, and Norway, the first auxilliums were mandatory for high school students; in France, it was mandatory for primary and secondary school students. In Spain [3,4], first aid appears as part of the curriculum for students in primary education (6–12 years) and in secondary education, and, in most cases, it is given within the subject of physical education [5]. However, Spanish teachers at the early childhood and primary education stages do not include specific training in first aid in their official state-level university curriculum [6,7]. This causes FA training among future teachers to be unequal in the Spanish territory. In fact, in 2019, less than 30% of university degrees in primary and/or early childhood education in Spanish public or certified universities included first aid in their curricula [8], which generates a debate on whether this training should be mandatory for all future teachers [9].

Special consideration for early childhood and primary education teachers is that they are the adults and first responders in the event of an accident or Unintentional Injury at school [3,10]. European records confirm that unintentional injuries (UI) and accidents suffered by children under 10 years of age occur mainly at home and at school [11]. In Spain, several studies have shown that most NTIs occurring at school happen during recess or during physical education classes and are usually traumatic in origin [12,13] (blows or blows, falls, and bad gestures) with a mild to moderate severity; the most frequent being contusions, wounds, bleeding, sprains and fractures. In 70% of cases, they require specific attention. In addition, Cardio Respiratory Arrest (CRA) can occur anywhere, and in any situation and in the pediatric population, sudden death and respiratory arrest are the main causes [14]. In this critical situation, early and adequate care by witnesses and the initiation of cardiopulmonary resuscitation, and an early call to emergency services increase survival and/or reduce possible sequelae.

To this, it should be added that, according to several studies [15], in general, teachers have difficulties preparing and conducting health education classes for students since they do not see the relevant support, which becomes a gap in this area [16,17,18]. This further highlights the importance of adequately training and educating teachers in this matter and in initiating specific programs in schools [19].

Therefore, several international [20,21,22] and national studies [23,24,25] where it is stated that first aid is the first care and immediate assistance given to a child when he or she has an injury, and care is taken with what is available at the time, hence how important it is to have the basic knowledge to provide help; that is why we have assessed the degree of knowledge and perceived ability of teachers to apply FA, concluding that most teachers do not feel adequately trained or competent to deal with these situations [15,16].

This highlights the importance of having an instrument that detects what knowledge teachers have about FA, how trained and skilled they feel, how important they consider this training to be for their profession, and how they demand to be trained in this regard.

Most of the studies carried out in Spain on this subject have used questionnaires [26,27,28,29] based mostly on objectives proposed by the ERC (European Resuscitation Council) or the AHA (American Heart Association), with closed-answer questions, with a single true option (sometimes assessing the degree of certainty in the response) [27] and, for the most part, applied to a sample limited to a locality or province.

To this analysis of instruments, it should be added that, according to recent qualitative research [30] carried out among student teachers in the Spanish autonomous city of Melilla, they demand, among other things, training in FA adapted to their school context.

Based on this premise and previous research, the present study aims to offer another instrument to assess the attitude of teachers and future teachers towards knowledge of first aid and to validate its psychometric properties: reliability and validity [31,32,33], through the accumulation of evidence to support the use and interpretation of the scores obtained on the questionnaire itself. Therefore, its real objective is not to evaluate how the sample responds to the designed questionnaire but what scores the test/questionnaire itself obtains when applied and, how they can be interpreted, based on specific objectives [34].

Consequently, this study establishes the following specific objectives:

- To develop conclusions about the content validity of the designed instrument through the agreement of a panel of experts.
- To analyze the psychometric properties of the designed questionnaire in order to calculate its reliability and to approach its internal structure by means of the Exploratory Factor Analysis.

## 2. Materials and Methods

### 2.1. Procedure

This project has been developed in accordance with current privacy and data protection regulations, which implies that participants gave their un-formed consent for those responsible for the study to process their personal data in accordance with the provisions of Regulation (EU) 2016/679, of 27 April (GDPR), and Organic Law 3/2018, of 5 December (LOPDGDD).

Before accessing the university and teaching population, the research project was approved by the academic committee of the Doctoral Program in Educational Sciences of the University of Granada.

Participants were informed through electronic means about the objectives, purpose, and benefits of the research, as well as the commitment to anonymity. The questionnaire was completed online through the Google formularies application.

### 2.2. Design and Sample

In order to achieve the objectives of the study, an online ad hoc questionnaire was designed, and its psychometric properties were analyzed.

The target population of the study consisted of teachers and future teachers (final year students) in the early childhood and/or primary education stages in Spain. In order to obtain a representative sample, the online questionnaire was sent to 19 Spanish faculties and/or public and/or subsidized universities, distributed among the northern, central, and southern zones of Spain (northern zone: including the Autonomous Communities of Galicia, Asturias, Cantabria, Basque Country, Navarre, Aragon, Catalonia, Castile and Leon, and La Rioja; central zone: Extremadura, Madrid, Castile-La Mancha, and Valencia; southern zone: Andalusia, Murcia, Autonomous Cities of Ceuta and Melilla and the Balearic and Canary Islands). The questionnaire was distributed through e-mails published on the official web pages of the various Faculties and, in more than 700 pre-school and/or primary schools in the same localities of the selected Faculties or Universities, through the public contact addresses that appear on the web pages of the Ministry on its page of the State Register of Non-University Teaching Centers. However, participation was not equally represented in all provinces or Autonomous Communities, so, finally, the sample consisted of 392 participants, of whom: 29% corresponded to university students in the last year of their infant and/or primary education degrees from Melilla (southern area of Spain), Madrid (central area of Spain), and Zamora (northern area of Spain) and 71% corresponded to active teachers in the infant and/or primary education stages, from schools in Aragon, Catalonia, Galicia, the Basque Country and Navarre (corresponding to the northern area of Spain), Madrid (corresponding to the central area of Spain) and Melilla, Andalusia, Murcia, the Balearic Islands and the Canary Islands (corresponding to the southern area of Spain).

Following the recommendations of Lloret-Segura [35], our study (with 392 participants) exceeded the minimum sample size required (200 cases).

In the sample, the mean age of the participants among active teachers was 40.5 years, with a distribution of 87.7% women and 12.3% men. The mean age of the participants among university education students was 22.3 years, with a distribution of 77% women and 23% men.

### 2.3. Instrument

The questionnaire on Attitude towards knowledge of first aid for teachers and future teachers consisted of sociodemographic questions and other specific questions on first aid. Specifically, 5 sociodemographic questions for student teachers (sex, age, university degree they were studying, faculty/university, and province of said faculty) and 6 sociodemographic questions for active teachers (sex, age, studies they had, years of experience, stage where they were teaching and province of their current school). The rest of the questionnaire was common, with 34 specific questions on first aid, grouped into 3 blocks or dimensions:

Block 1 consisted of 8 questions on previous first aid experience, valuing its importance and training demanded; Block 2 consisted of 21 questions on evaluation of attitude towards FA knowledge and FA preparedness for a school setting; Block 3 consisted of 5 questions on self-assessment of own knowledge and skills.

Each question in block 2 contained 4 possible answers, each of which was to be rated according to the degree of agreement with the described performance, in Disagree (D), Partially Agree (PA), Agree (A), or Strongly Agree (TA). The pre-questions in block 3 were all dichotomous with a yes/no response.

The questions were adapted from the questionnaires of López-Felpeto, Navarro-Patón and Basanta-Camiño [26], Abraldes and Ortín [27], the pre-course evaluation of basic CPR and AED offered by the Spanish Society of Intensive Care Medicine, Critical Care and Coronary Units (Semicyuc) [28] and Miró et al. [29]. Other questions were also added based on the review and investigation of the most frequent events in the school setting and the most serious or worrisome for teachers (loss of consciousness, choking, allergies, DM disorders, seizures, asthmatic crisis...) [19,29,30].

### 2.4. Data Analysis

To assess the correct design of the questionnaire, its content validity, reliability, and construct validity were evaluated. Various psychometric assessment strategies and tools were used for this purpose.

In order to assess content validity, the objective was to determine whether the questionnaire reflected a specific domain of first aid content (i.e., whether the selected items included all relevant aspects of first aid in the school setting). For this purpose, expert consultation [36,37] was used to identify the degree of adequacy, relevance, and congruence of each of the questions in the questionnaire. An estimation scale with a degree of agreement of 1–3 was designed for each of the questions in the questionnaire, and, in this way, the most reliable consensus opinion could be obtained (avoiding the decision falling on a single professional) [38,39,40]. In addition, the opportunity was offered to make suggestions for each question by means of an open response section [41].

For this purpose, in the first stage, the research group selected 7 experts in the subject matter, consisting of: 5 physicians (4 of them specialists in Family Medicine and 1 of them specialist in Internal Medicine, all of them with more than 10 years of experience) and 2 University professors of education degrees, specialists in research methodology, with experience of more than 10 years as university teachers. All of them were asked for their consent to participate and were distributed via e-mail the initial questionnaire, which included a brief introduction defining first aid, indicating the main objective, and the instructions for filling it in. A total of two rounds were carried out (with a difference of 14 days between them), in which aspects of wording or type of response were modified. In a second stage and according to the previous recommendations, the final version of the questionnaire was developed, and the degree of agreement of each question was analyzed, considering adequate values of those >80% to be able to maintain the question.

The aim was to determine the accuracy and homogeneity of the questionnaire items. For this purpose, Cronbach’s alpha coefficient to assess reliability [31,42,43] (based on the average correlation between all the items of a test), whose values range from 0–1, was used. For these to be considered acceptable, they must be >0.7.

Finally, to assess construct validity [44], the aim was to approach the empirical, theoretical model underlying the variable of interest (attitude and degree of agreement with knowledge in BP) and to identify the number of common factors necessary to identify the common variance of the set of items analyzed. The technique used was Exploratory Factor Analysis (EFA).

The recommendations of Lloret-Segura [35] on Exploratory Factor Analysis (EFA) were taken into account:

- It should be consistent with the nature of the items, using appropriate software. In this case, polytomous items [45] (with Likert scale according to degree of agreement: D, PA, A, TA) and the SPSS 25.0 AMOS V.25.0 program were used.
- The adequacy of the data should be checked at least by means of the KMO test (which relates the correlation coefficients observed between the variables). It would be adequate with values between 0.7–0.79 and satisfactory with values > 0.8. Bartlett’s test is considered acceptable with a significance (*p*-value) < 0.05.

After the EFA results, the number of adequate factors was generated and identified, the items whose results were not satisfactory were eliminated, and the remaining items were assigned to each factor. In this way, the questionnaire was re-structured.

## 3. Results

With regard to content validity (carried out by the panel of experts), of the 39 final items used in the questionnaire, five of them were initially modified in the style of writing, one question was added (related to allergies) and block two (attitude towards knowledge in BP) was re-structured, to replace a single valid response with evaluations of the degree of agreement of each possible response (D, PA, A, TA) and thus increase the descriptive information on what they know and decrease the participants’ feeling of evaluation. The remaining items were not modified.

After these modifications and after the second round, the concordance in all the questions was >0.8, so they were all maintained in the questionnaire.

Regarding reliability, the result obtained was a Cronbach’s alpha of 0.773, which was considered “acceptable” reliability.

As for construct validity, Exploratory Factor Analysis obtained the following values:

The sedimentation plot (see Appendix A) showed that the first four factors explained most of the total variability in the items, with values greater than one. After the initial analysis, three factors were finally selected.

The sample adequacy measures [35,45,46] to check if the data are appropriate for factor analysis, obtained a KMO (Kaiser–Meyer–Olkin) test with a value of 0.804 (acceptable); and Bartlett’s test of sphericity of: 1471.332 (gl: 378; *p*-value < 0.001), suggesting that the variables analyzed are correlated in the sample, and the data can be subjected to factor analysis [47].

Through the rest of the analysis, it was obtained that 32.86% of the total variance can be explained through the three factors previously identified.

Table 1 shows the descriptive statistics of the questionnaire items, through which it is observed that in most of the items (>67%), a degree of agreement predominates in the answers between DA-TA (agree-totally agree), except in the last ones (items 30–34, with dichotomous yes/no answers, whose data show a tendency towards the self-assessment of not possessing the necessary skills). As indicated by the skewness and kurtosis indices, the questionnaire items tend toward a non-normal distribution.

For the analysis of the correlation structure, an orthogonal factor rotation was performed (Varimax method with Kaiser normalization). This was used to determine which group of variables had a high correlation with each factor, the criterion for inclusion being a factor saturation >0.3 [46,48]. Thus, three items (items 10, 13, and 26) were eliminated as they had lower saturation values. These questions were related to the following topics: bleeding, sprains, and CPR compression/ventilation parameters, respectively). The rest of the items could be associated with each of the four factors identified, being associated with Factor 1, items: 7, 8, 12, 14, 14, 15, 18, 19, 20, 21, 22, 24, 28, 29. To Factor 2, items: 9, 11, 16, and 25. To Factor 3, items: 23, 30, 31, 32, 33, 34; and to Factor 4, items: 17 and 27. Since Factor 4 only included two items, this factor was eliminated, and its items were reassigned to the next factor with which it presented greater saturation (specifically, Factors 1 and 2, respectively). See Table 2 and Table 3.

After that, the factors were named, and the questions were redistributed according to the association with these factors.

Factor 1: Attitude towards general knowledge in FA and learning methodology (This factor works on aspects that range from how to act in the event of a slight contusion, what to do in the event of a fracture, a burn, with diabetic and asthmatic children)

Factor 2: Attitude towards wounds and CPR algorithm (most frequent and/or severe events) (The action before bleeding wounds, shortness of breath, and how to perform cardiac massage is analyzed here)

Factor 3: Self-perception of knowledge or skill in FA (Knowing how to act in a more technical way, such as the use of a defibrillator and giving an injection).

Finally, Pearson’s correlation coefficient was applied to determine the percentage of shared variance between the three factors [49,50]. Table 4 shows that the highest correlations are between Factors 1 and 2, which have a covariance of 0.422, this relationship being significant (with a *p*-value < 0.01) and considered “moderate”; as well as Factors 1 and 3, with a covariance of 0.244 (also a significant association with *p*-value < 0.01), considered a positive co-relation but “low”.

**Figure 1:**
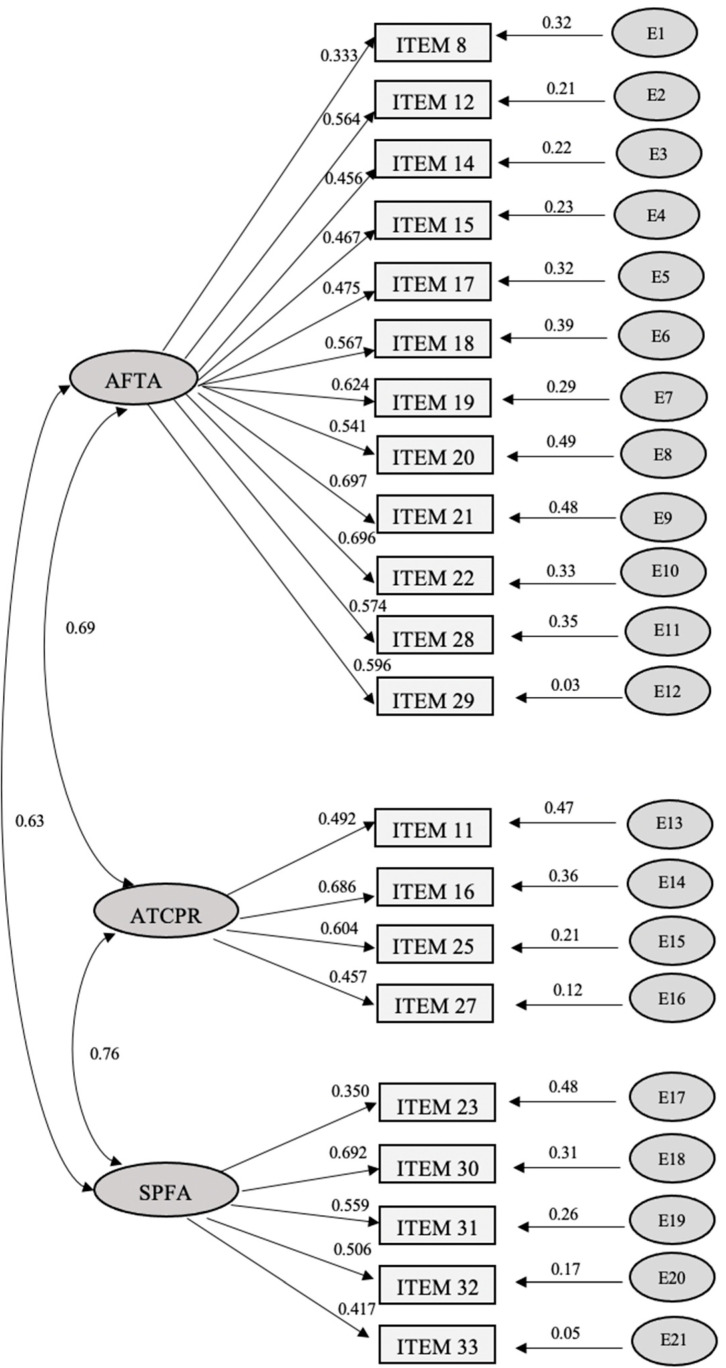
Structural equation model for Attitude towards first aid Knowledge Questionnaire (AFAK-Q).

Next, the proposed model of the exploratory analysis was confirmed by means of confirmatory factor analysis (using a structural equation model, see Figure 1).

Items 7, 9, 24, and 34 were eliminated to improve the fit. The proposed model was readjusted to three factors, with the following fit indices: RMSEA (Root Mean Square Error of Approximation), CFI (Comparative Fit Index), and NFI (Normed Fit Index). The results of these indices were: RMSEA of 0.043 (LO90 = 0.037; HI90 = 0.051) considered adequate; CFI of 0.924, very close to the target value of 0.95; NFI 0.911 (acceptable). In graph one of the structural equation, the relationship between the three factors can also be observed, showing how the strongest relationship occurs between Factors 2 and 3 (0.76).

In order to relate and predict the value of the dependent variable “general knowledge of first aid in teachers and future teachers” with respect to the value of the selected explanatory variables (sex, age, degree of importance attributed to FA, consideration of the level of knowledge in FA, consideration of the level of preparation to act correctly applying FA), Factor 1: attitude towards general knowledge in first aid and learning methodology (AFTA), Factor 2: attitude towards wounds and cardiopulmonary resuscitation (ATCPR), and Factor 3: self-perception of knowledge or skill in first aid (SPFA), a multivariate analysis of multiple linear regression stepwise was performed. To this end, linearity (by means of a partial scatter plot), independence of errors (by means of the Durbin–Watson statistic, considering appropriate values between 1.5–2.5), homoscedasticity (by means of the scatter plot and Levène test with *p*-value > 0.05), normality of the variables (by means of a histogram) and non-collinearity (with a tolerance >0.10 and an IVF (variance inflation factor) <10) were checked. The variables considered predictors that were selected based on a *p* < 0.05 in the t-test columns and their weights according to their beta-typed coefficients.

According to the results shown in Table 5, three models emerged from the regression analysis. Model 1 offered the greatest explanatory power since it explained the greatest percentage of the variance (see the corrected *R*^2^ column in Table 5).

After obtaining these appropriate values, the model was interpreted, where it was observed that, of all the variables included in the model, only the one corresponding to Factor 1 “AFTA” had a probability of error *p*-value < 0.05 for the t value, and was, therefore, the only variable that could be considered as a predictor. This variable presents a high beta type coefficient with a positive value, which indicates its positive trend; that is, the higher the score on the questionnaire items included in Factor 1 “AFTA” variable, the greater the general knowledge of BP. In addition, this model explains 68.1% of the variance of the dependent variable significantly, as obtained in the significance (*p*-value < 0.05) of the ANOVA test.

## 4. Discussion

The aim of the present study was to analyze and validate the content of an instrument designed to analyze the attitude towards first aid knowledge and skills of teachers and future teachers in Spain, specifically for the school context. From the results obtained, we can answer that its design is acceptable, as shown by the tests carried out on its content validity, reliability, and construct validity, based on the required parameters [34,35,46].

Several questionnaires [26,27,29] were validated for smaller populations (confined to the same province, Autonomous Community, or the same area of the national territory) and which, in most cases, focused on the recognition of a correct action for the adequate application of first aid were taken into account. The main novelties of the present study with respect to the previous ones are: the contextualization in a school environment, the intention of directing its application to the entire national territory, and the evaluation of the degree of agreement with each possible response providing a more descriptive approach.

Finally, the questionnaire was validated using the panel of experts (M. Delphi), Cronbach’s reliability index, and Exploratory Factor Analysis. Based on the results obtained, the questionnaire has undergone, since its initial planning, modifications in the style of writing and type of responses; items have been eliminated and regrouped according to the degree of correlation with the factors identified. Specifically, three main factors have been recognized as influencing the variance of the items dependent on the same factor, these being: attitude towards general knowledge in FA and learning methodology, attitude towards injuries and CPR algorithm (most frequent and most serious events), and self-perception of knowledge or skills in FA. This could respond to two hypothetical latent factors: training and experience in first aid applied to the school environment; and self-perception and reflection on the ability to apply FA and act in case of need. Both are aspects highlighted in other studies, where teachers demand specific training in FA and recognize them as useful training for their profession [9,19].

On the other hand, a moderate correlation [50,51] was found between the factors “attitude to general knowledge in FA and learning methodology” and “attitude to injuries and CPR algorithm (most frequent and/or serious events)”. This relationship can be explained by the need that teachers request and show to be trained adequately and in a practical manner and adapt to their school context, in first aid and, especially in Cardio Pulmonary Resuscitation maneuvers, where they show the greatest deficits. This coincides with the recent studies already mentioned by Llorent and Cobano [25], Abelairas et al. [23], and Navarro et al. [24].

Although with a low correlation, an association was also found between the factors “attitude towards general knowledge in FA and learning methodology” and “auperception of knowledge or skill in FA”, from which it can be inferred that the degree of preparation and knowledge in FA causes the individual to be more aware of his or her limitations and skills, which could be related to safety and pre-disposition to act [16].

However, certain limitations that were noted during this validation process should be mentioned. One of them was that the recruitment of the sample was not equally represented among all the zones of the Spanish territory, so the northern zone was the least represented. In addition, the initial selection of four factors was later reduced to three since the fourth factor included only two items of the questionnaire, and these could be more coherently included within the content of Factors 1 (item 17) and 2 (item 27), even though in this case, the saturation of item 17 was close to the lower limit required, being 0.293; there is also the fact that, in this case, the saturation of item 17 was close to the lower limit required: 0.293; it should also be noted that three items had to be deleted (for not reaching acceptable values in the rotated component matrix). Our interpretation is that this may have been due to three reasons: poor understanding of the question or of the answers (due to inadequate writing style or use of overly technical vocabulary), broad ignorance of the subject matter of the question (requiring specific training to be able to answer, which leads to dispersion in the response), difficulty for the participant to discern between appropriate actions and inappropriate actions that are widely disseminated or frequently performed by the general population. Here lies part of the importance of training teachers in first aid in a generalized manner so that they can have a safe and correct attitude when identifying and applying appropriate actions and avoiding those that are inappropriate in first aid within the school environment.

In addition to the limitations described above, it is necessary to highlight the following with respect to possible studies that could lead to further improvements in the future. First, the sample should be expanded to include a more representative sample from Spain (and perhaps Europe). Likewise, the need for more studies capable of providing evidence on the competencies in Immediate Health Care Measures at School by teachers is highlighted. It would be useful to include specialists in medicine or nursing to bring this concept to life, both in teacher training and in recycling seminars for practicing teachers. Another limitation faced by the present study was the difficulties of access to the sample caused by the SARS-CoV 2 virus, which complicated the participation of the respondents since, both in the universities and in the schools, there were adaptations to blended learning during the recruitment course of the sample, and the complexity was doubled. Nevertheless, it is important to point out that data collection was completed as established, only in a longer period of time, and this increase was acceptable.

For all these reasons, and as proposals for improvement, it is proposed to increase the sample size, ensuring a more equitable origin of the national territory in the sample, as well as to carry out a subsequent Confirmatory Factor Analysis of the data obtained.

## 5. Conclusions

Schoolchildren spend an average of 6–7 h a day in Spanish schools from 3 to 16 years of age, with qualified personnel in various subjects; however, our study concludes that they do not have the necessary knowledge to intervene in cases of Immediate Health Measures at School. Our research has shown that the questionnaire developed has adequate psychometric properties, and with our regression results, we have demonstrated the importance of including first aid training in teachers’ qualifications. We consider that these needs could be easily met by means of practical first aid intervention seminars, as well as the inclusion of a subject in teacher training plans. Based on this research, we would like to highlight the importance of this training in Immediate Health Care and call on the authorities and the general public to include first aid knowledge for teachers and future teachers in seminars, lectures, or in the training curricula.

## Figures and Tables

**Table 1:** Descriptive statistics of the items of the first aid questionnaire in school context: mean, standard deviation, skewness and kurtosis.

Item	$\overset{—}{X}$	SD	Asymmetry	Kurtosis
7	3.192	0.912	−0.874	−0.193
8	3.813	0.457	−2.777	9.187
9	2.550	1.090	−0.165	−1.269
10	2.490	1.083	0.023	−1.273
11	2.855	1.093	−0.502	−1.067
12	3.521	0.752	−1.711	2.672
13	2. 372	1.177	0.133	−1.481
14	3.404	0.801	−1.384	1.494
15	3.198	0.947	−0.977	−0.048
16	3.288	0.826	−1.089	0.718
17	3.171	0.936	−0.912	−0.130
18	3.516	0.759	−1.664	2.419
19	3.777	0.549	−2.516	6.055
20	3.449	0.810	−1.442	1.481
21	3.490	0.748	−1.547	2.107
22	3.702	0.608	−2.334	5.831
23	2.743	1.153	−0.254	−1.410
24	3.763	0.595	−2. 873	8.602
25	3.387	0.860	−1.236	0.575
26	2.036	1.052	0.577	−0.956
27	3.243	1.008	−1.110	−0.006
28	3.422	0.803	−1.329	1.096
29	3.605	0.717	−1.913	3.222
30	1.464	0.499	0.170	−1.981
31	1.719	0.455	−0.927	−1.147
32	1.304	0.457	0.899	−1.198
33	1.512	0.501	−0.041	−2.009
34	1.371	0.482	0.562	−1.693

**Table 2:** Rotated component matrix of the items of the first aid in school context questionnaire.

Variables	Rotated Factorial Matrix				Rotated Factorial Matrix (Omitted the Lower Charges to 0.300)			
	F1	F2	F3	F4	F1	F2	F3	F4
ITEM 7	0.543	−0.216	0.111	−0.119	0.543			
ITEM 8	0.617	−0.214	0.075	−0.132	0.617			
ITEM 9	−0.113	0.480	0.086	−0.061		0.480		
ITEM 10 ^1^	0.050	0.257	−0.202	0.223				
ITEM 11	0.101	0.721	0.091	0.014		0.721		
ITEM 12	0.437	0.358	−0.102	0.028	0.437	0.358		
ITEM 13 ^1^	0.035	0.193	−0.151	−0.647				
ITEM 14	0.323	0.125	−0.009	0.274	0.323			
ITEM 15	0.413	0.224	0.195	0.270	0.413			
ITEM 16	0.321	0.571	0.078	−0.025	0.321	0.571		
ITEM 17	0.293	0.269	−0.126	0.313				0.313
ITEM 18	0.534	0.264	−0.050	0.293	0.534			
ITEM 19	0.687	0.077	−0.017	0.081	0.687			
ITEM 20	0.544	0.091	0.113	−0.099	0.544			
ITEM 21	0.589	0.315	−0.031	0.297	0.589	0.315		
ITEM 22	0.629	0.176	0.082	0.043	0.629			
ITEM 23	0.222	−0.014	0.326	−0.221			0.326	
ITEM 24	0.567	0.106	−0.086	0.121				
ITEM 25	0.323	0.475	0.245	−0.176	0.323	0.475		
ITEM 26 ^1^	0.001	0.167	−0.199	−0.577				
ITEM 27	0.236	0.321	0.091	0.324		0.321		0.324
ITEM 28	0.442	0.386	0.176	−0.167	0.442	0.386		
ITEM 29	0.572	0.054	0.104	0.106	0.572			
ITEM 30	0.081	0.197	0.706	0.103			0.706	
ITEM 31	0.236	0.197	0.568	0.270			0.568	
ITEM 32	−0.049	0.008	0.720	0.080			0.720	
ITEM 33	−0.028	0.260	0.445	0.049			0.445	
ITEM 34	0.009	−0.314	0.479	0.037			0.479	

^1^ Items eliminated for not reaching saturation > 0.3.

**Table 3:** Assignment of variables to the 3 factors. Factor loading of AF3 dimensions.

Variables	F1	F2	F3
Item 7	0.543		
Item 8	0.617		
Item 12	0.437		
Item 14	0.323		
Item 15	0.413		
Item 17	0.293		
Item 18	0.534		
Item 19	0.687		
Item 20	0.544		
Item 21	0.589		
Item 22	0.629		
Item 28	0.442		
Item 29	0.572		
Item 9		0.480	
Item 11		0.721	
Item 16		0.571	
Item 25		0.475	
Item 27		0.321	
Item 23			0.326
Item 30			0.706
Item 31			0.568
Item 32			0.720
Item 33			0.445
Item 34			0.479
Alfa (0.773)			

**Table 4:** Correlation of factors.

	Factor 1	Factor 2	Factor 3
Factor 1: Attitude towards general knowledge in FA and learning methodology	1		
Factor 2: Attitude towards wounds and CPR algorithm (most frequent and/or severe events)	0.422 **	1	
Factor 3: Self-perception of knowledge or skill in FA	0.244 **	0.225 **	1

** The correlation is significant at the 0.01 level (bilateral).

**Table 5:** Stepwise Multiple Linear Regression Model for predicting (AFAK-Q).

Model ^d^	*R*	*R* ^2^	*R*^2^ Corrected	Standard Error	*F*	*p* de *F*	Dubin-Watson
1	0.835 ^a^	0.697	0.681	0.182	44.315	<0.001	2.207
2	0.747 ^b^	0.558	0.535	0.219	24.319	<0.001	
3	0.444 ^c^	0.197	0.155	0.297	4.694	<0.001	

(^a^) Constant, gender, age, educational stage, self-perceived level of knowledge in FA, self-perceived level of preparedness to act in FA, degree of importance and Factor 1 AFTA. (^b^) Constant, gender, age, educational stage, self-perceived level of knowledge in FA, self-perceived level of preparedness to act in FA, degree of importance and Factor 2 ATCPR. (^c^) Constant, gender, age, educational stage, self-perceived level of knowledge in BP, self-perceived level of preparedness to act in BP, degree of importance and Factor 3 SPFA. (^d^): Dependent variable: general first aid knowledge.

## Data Availability

Not applicable.

## Appendix A

### Template of the Administered Questionnaire for Attitude towards First Aid Knowledge Questionnaire (AFAK-Q)

**Indicate what is your option:** Student Teachers Active Teachers				
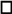
**Sex**: Mujer Hombre				
**Age:** ______				
**University degree they were studying faculty/university:**				
Early Childhood Education Degree				
Degree in elementary education				
Double Degree in Early Childhood Education and Primary Education				
Double Degree in Primary Education and Physical Activity and Sports Sciences				
Others___________________________________________________________				
**Name of faculty/university: _____________________________________________**				
**Province: ______________________________________________________________**				
**If you are an Active Teacher, years of experience: ____________________________**				
**SPECIFIC QUESTIONS ABOUT FIRST AID**				
1. Have you previously taken specific first aid courses? Yes No				
2. If so, where did you do it?				
In a specific subject of First Aid of the University Curriculum				
In a part of a subject of the University Curriculum				
Through conferences, congresses from the University				
Through official training outside the University Degree				
Through a course from an external entity				
3. How long ago did you receive this training with content in First Aid?				
Less than 1 year ago				
1–2 years ago				
3–5 years ago				
More than 5 years ago				
From 1 to 4, with 1 being nothing and 4 being quite, following questions:				
**ITEMS**	**Nothing** **(1)**	**Little** **(2)**	**Enough** **(3)**	**Quite** **(4)**
4. How do you consider your KNOWLEDGE LEVEL in First Aid				
5. How do you consider your level of PREPARATION TO ACT CORRECTLY in a situation that requires first aid				
6. Importance that you attribute to first aid for your work or future work as a teacher.				

Rate from 1 to 4 the degree of importance that you attribute to first aid for your work or future work as a teacher.				
**ITEMS**	**Disagree** **(1)**	**Partially Agree** **(2)**	**Agree** **(3)**	**Total Agree** **(4)**
8: Do you think that the methodology used to train you in First Aid should be based on face-to-face classes with practical cases?				
11. If a student receives a blow to the nose and it starts to bleed, should we compress the wing of the nose of the office where it bleeds for 5–7 min?				
12. When there is a MILD CONTUSION (blow or “bump”), should local cold be applied?				
14. Faced with a student who, after a fall, complains of a lot of pain in a limb, cannot move it and also seems to be deformed, do you think we should not move it?				
15. A 10-year-old boy collides with another boy and, as a result, a tooth falls out on the floor and bleeds from the mouth. Would you tell him to rinse with very cold water and press down on the gum by biting down on a piece of sterile gauze? (in addition to calling the family to take him urgently to the dentist)				
16. If a student accidentally suffers an amputation (for example, of a finger), would you press the wound with gases or clean cloths until 112 arrived?				
17. In the event of a heat burn, during the first few minutes, should we keep the area under running water for at least 5–10 min?				
18. In the event of a seizure, is it important to control the duration of the seizure while 112 arrives?				
19. If a known diabetic student begins to “feel bad”, with weakness or intense irritability, dizziness, confusion, paleness, sweating, nausea … and we cannot differentiate between very high levels of sugar or very low levels, we must call to 112 and to the family.				
20. You are a physical education teacher. In the middle of the class, one of the girls who was running, begins with an intense cough and shortness of breath. She tells you that she is asthmatic and that she has the “VentolínR” in her backpack. At the beginning of the course, the parents informed you that their daughter knows how to use said medication and that she needs it on occasion, they also provided you with a written medical report indicating that she may need this medication during exercise. Would you tell her to self-administer VentolínR?				
21. During Physical Education class on a hot day, while the students are running in the playground, one of them falls to the ground (passes out), without moving or getting up. When you come running to his side, he has just opened his eyes and is able to sit up, but he tells you that he is a little dizzy. Would you keep him lying down with his legs elevated, preferably in a shady place, until they took him away?				
22. You are in the schoolyard and suddenly two children come running to tell you that another student is lying on the ground and does not react or get up. If he doesn’t answer when I touch or call him, I would check if he is breathing and call 112				
23. To find out if a person is breathing, would you uncover the chest and see if it moves?				
25. Should we call 112 and the family if a 6-year-old student is unconscious on the floor and we have verified that he is NOT breathing?				
27. Should the hands be positioned in the center of the chest, on the lower half of the sternum, to perform the cardiac massage?				
28. You are at breakfast time and one of your students (who was eating a ham sandwich) starts coughing non-stop, to the point that the cough becomes weak, he cannot speak and puts his hands around his neck. Would you alternate 5 back snaps and 5 abdominal thrusts (Heimlich)?				
29. If a person who is choking, loses consciousness. Would you start CPR (heart massage and breaths)?				
Note: Items 7,9,10,13,24 and 26 have been eliminated, but the original numbering has been left for a better understanding of the established grouping of factors.				

**ITEMS**	**YES**		**NO**	
30. Would you know how to perform the head-chin maneuver to keep the airway open (and facilitate the passage of air)?				
31. Do you know what a Semi-Automatic External Defibrillator is? (DESA)				
32. Would you know how to use it and follow its instructions correctly? Deleted				
33. Faced with a child who suffers an allergic reaction and has his injectable, would you know how to apply it?				
Note: Item 34 has been eliminated.				
